# Supplementary material for: Characterization and In Vitro Biocompatibility of Two New Bioglasses for Application in Dental Medicine—A Preliminary Study
Source: Materials (Basel). 2022 Dec 18;15(24):9060. doi: 10.3390/ma15249060 (PMC9782195; doi:10.3390/ma15249060)
Supplement: Supplementary file 1 [file materials-15-09060-s001.zip › materials-2027281-supplementary.pdf]

Supplementary Materials

Characterization and In Vitro Biocompatibility of Two New Bioglasses for Application in Dental Medicine—A Preliminary Study

Andra Clichici [1], Gabriela Adriana Filip [2,*,†], Marcela Achim [3,†], Ioana Baldea [2], Cecilia Cristea [4], Gheorghe Melinte [4], Ovidiu Pana [5], Lucian Barbu Tudoran [5], Diana Dudea [1] and Razvan Stefan [6]

[1] Department of Propaedeutics and Dental Materials, Faculty of Dental Medicine, Iuliu Hatieganu University of Medicine and Pharmacy, 400012 Cluj-Napoca, Romania
[2] Department of Physiology, Iuliu Hatieganu University of Medicine and Pharmacy, 400006 Cluj-Napoca, Romania
[3] Departments of Pharmaceutical Technology and Biopharmaceutics, Iuliu Hatieganu University of Medicine and Pharmacy, 400606 Cluj-Napoca, Romania
[4] Analytical Chemistry Department, Faculty of Pharmacy, Iuliu Haţieganu University of Medicine and Pharmacy, 400349 Cluj-Napoca, Romania
[5] National Institute for Research and Development of Isotopic and Molecular Technologies, 400293 Cluj-Napoca, Romania
[6] Faculty of Veterinary Medicine, University of Agricultural Sciences and Veterinary Medicine, 400372 Cluj-Napoca, Romania

Material and Methods

Preparation and characterizations of the BGs

The analysis of solid BG composition before immersion was performed by XPS analysis and X-Ray diffractometry (XRD). XRD was conducted using a Shimadzu 600 XRD diffractometer (Shimadzu Corporation, Kyoto, Japan) with voltage of 40 kV and a current of 30 mA. All samples were crushed into fine powders and scanned from 10–60 theta degree.

Results

XPS measurements and XRD analysis

In order to obtain information about the chemical compositions before immersion, X-ray photoelectron spectroscopy (XPS) measurements were performed on the powdered samples of two BGs. For better correlation between sample composition and their behavior in biological fluids, the ratios of atom pairs were calculated, as listed in Table S1 (Supplementary Files).

Table S1. Ratio between different atoms in the considered samples.

	BG1	BG2
B/Ca	2.33	2.36
B/F	6.80	10.06
Ca/F	2.91	4.24
V/B	0.002	0.004
V/Ca	0.005	0.010

Through XRD, no crystalline phases were detected, the diffraction patterns being large and noisy, characteristic of vitreous and homogeneous samples (Supplementary Materials, Figure S1).

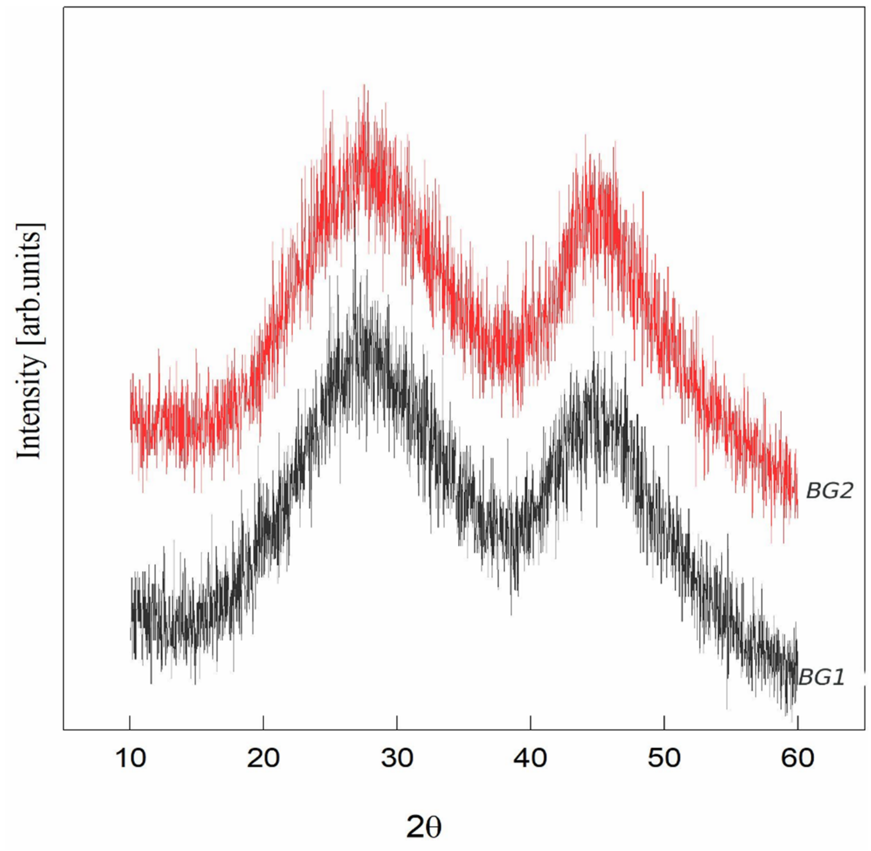

Figure S1. XRD diffraction patterns of BG1 and BG2 samples before immersion in PBS.
